# Ethyl 2-(2-methyl-4-nitro-1*H*-imidazol-1-yl)acetate

**DOI:** 10.1107/S1600536810037098

**Published:** 2010-09-25

**Authors:** Hong-Yong Wang, Pei Zou, Min-Hao Xie, Yong-Jun He, Jun Wu

**Affiliations:** a Key Laboratory of Nuclear Medicine, Ministry of Health, Jiangsu Key Laboratory of Molecular Nuclear Medicine, Jiangsu Institute of Nuclear Medicine, Wuxi 214063, People’s Republic of China

## Abstract

In the title compound, C_8_H_11_N_3_O_4_, the dihedral angle between the imidazole ring and the ethyl acetate plane is 103.1 (8)°. The crystal packing is stabilized by weak inter­molecular C—H⋯O and C—H⋯N hydrogen bonds.

## Related literature

For the possible use of nitro­imidazole derivatives as radio sensitizers, to enhance the lethal effect of ionizing radiation on hypoxic tissues, see: Brown (1989 ▶); Chapman (1979 ▶); Chu *et al.* (2004 ▶).
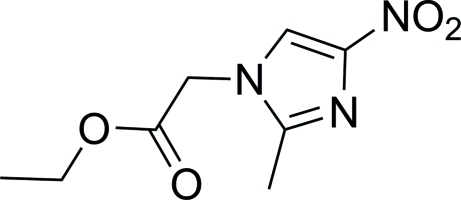


## Experimental

### 

#### Crystal data

C_8_H_11_N_3_O_4_
*M* *_r_* = 213.20
Orthorhombic, 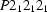

*a* = 4.416 (3) Å
*b* = 10.290 (6) Å
*c* = 20.769 (12) Å
*V* = 943.7 (10) Å^3^
*Z* = 4
Mo *K*α radiation
μ = 0.12 mm^−1^
*T* = 103 K
0.53 × 0.53 × 0.18 mm

#### Data collection

Rigaku SPIDER diffractometer
7883 measured reflections
1306 independent reflections
1161 reflections with *I* > 2σ(*I*)
*R*_int_ = 0.037

#### Refinement

*R*[*F* ^2^ > 2σ(*F* ^2^)] = 0.033
*wR*(*F* ^2^) = 0.082
*S* = 1.00
1306 reflections
138 parameters
H-atom parameters constrained
Δρ_max_ = 0.24 e Å^−3^
Δρ_min_ = −0.20 e Å^−3^

### 

Data collection: *RAPID-AUTO* (Rigaku, 2004 ▶); cell refinement: *RAPID-AUTO*; data reduction: *RAPID-AUTO*; program(s) used to solve structure: *SHELXS97* (Sheldrick, 2008 ▶); program(s) used to refine structure: *SHELXL97* (Sheldrick, 2008 ▶); molecular graphics: *SHELXTL* (Sheldrick, 2008 ▶); software used to prepare material for publication: *SHELXTL*.

## Supplementary Material

Crystal structure: contains datablocks I, global. DOI: 10.1107/S1600536810037098/pv2327sup1.cif

Structure factors: contains datablocks I. DOI: 10.1107/S1600536810037098/pv2327Isup2.hkl

Additional supplementary materials:  crystallographic information; 3D view; checkCIF report

## Figures and Tables

**Table 1:** Hydrogen-bond geometry (Å, °)

*D*—H⋯*A*	*D*—H	H⋯*A*	*D*⋯*A*	*D*—H⋯*A*
C5—H5*A*⋯O4^i^	0.99	2.56	3.338 (3)	135
C5—H5*A*⋯N2^i^	0.99	2.56	3.509 (3)	160
C5—H5*B*⋯O2^ii^	0.99	2.39	3.175 (3)	136
C7—H7*A*⋯O2^iii^	0.99	2.46	3.362 (3)	151

Symmetry codes: (i) 
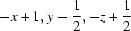
; (ii) 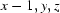; (iii) 
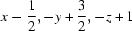
.

**Fig. 1.**
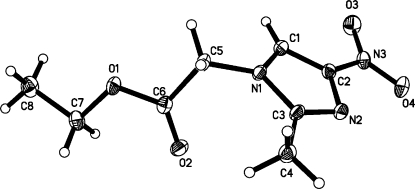
The molecular structure of the title compound, with the atomic numbering scheme. Displacement ellipsoids are drawn at the 50% probability level.

## supplementary crystallographic information

### Comment

Nitroimidazole derivatives have a tendency to be accumulated in the hypoxic regions leading to the possibility of envisaging these compounds as radio sensitizers, the agents which enhance the lethal effect of ionizing radiations on hypoxic tissues (Chapman, 1979; Brown, 1989; Chu *et al.*, 2004). As a contribution to this field, we present here the title compound, (I), synthesized by a simple and efficient method.

In (I) (Fig. 1), the imidazole group is essentially planar and forms a dihedral angle of 103.1 (8)° with the ethyl acetate plane defined by atoms C5–C8/O1/O2. The nitro group lies in the plane of the imidazole group. In the cystal structure, the packing is stabilized by weak C—H···O and C—H···N interactions.

### Experimental

The title compound was prepared by the following procedure. To a solution of 2-methyl-4-nitroimidazole (2.11 g, 0.01 mol) in ethyl 2-chloroacetate (14.2 ml, 0.1 mol), propionic acid (6.66 ml) was added and refluxed for 16 h. The mixture was filtered and concentrated under reduced pressure. The residue was purified by column chromatography (silica gel, ehtyl acetate/petroleum ether, 1:1). Single crystals were obtained by using ethanol/water (2:1) as solvents for recrystallization (m.p. 383–385 K).

### Refinement

The H atoms were positioned geometrically and allowed to ride on their parent atoms at distances C—H ═ 0.95, 0.98 or 0.99 Å for aryl, methyl and methylene H-atoms with *U*_iso_(H) ═1.2 *U*_eq _(parent atom). An absolute structure could not be established by anomalous dispersion effects in diffraction measurements on the crystal. Therefore, 858 Friedel pairs were merged.

### Crystal data

**Table:**

C_8_H_11_N_3_O_4_	*F*(000) = 448
*M**_r_* = 213.20	*D*_x_ = 1.501 Mg m^−^^3^
Orthorhombic, *P*2_1_2_1_2_1_	Mo *K*α radiation, λ = 0.71073 Å
Hall symbol: P 2ac 2ab	Cell parameters from 2529 reflections
*a* = 4.416 (3) Å	θ = 3.6–27.6°
*b* = 10.290 (6) Å	µ = 0.12 mm^−^^1^
*c* = 20.769 (12) Å	*T* = 103 K
*V* = 943.7 (10) Å^3^	Chunk, colorless
*Z* = 4	0.53 × 0.53 × 0.18 mm

### Data collection

**Table:**

Rigaku SPIDER diffractometer	1161 reflections with *I* > 2σ(*I*)
Radiation source: Rotating Anode	*R*_int_ = 0.037
graphite	θ_max_ = 27.6°, θ_min_ = 3.6°
ω scans	*h* = −5→5
7883 measured reflections	*k* = −13→13
1306 independent reflections	*l* = −27→27

### Refinement

**Table:**

Refinement on *F*^2^	Primary atom site location: structure-invariant direct methods
Least-squares matrix: full	Secondary atom site location: difference Fourier map
*R*[*F*^2^ > 2σ(*F*^2^)] = 0.033	Hydrogen site location: inferred from neighbouring sites
*wR*(*F*^2^) = 0.082	H-atom parameters constrained
*S* = 1.00	*w* = 1/[σ^2^(*F*_o_^2^) + (0.0463*P*)^2^ + 0.16*P*] where *P* = (*F*_o_^2^ + 2*F*_c_^2^)/3
1306 reflections	(Δ/σ)_max_ < 0.001
138 parameters	Δρ_max_ = 0.24 e Å^−^^3^
0 restraints	Δρ_min_ = −0.20 e Å^−^^3^

### Special details

**Table:**

Geometry. All e.s.d.'s (except the e.s.d. in the dihedral angle between two l.s. planes) are estimated using the full covariance matrix. The cell e.s.d.'s are taken into account individually in the estimation of e.s.d.'s in distances, angles and torsion angles; correlations between e.s.d.'s in cell parameters are only used when they are defined by crystal symmetry. An approximate (isotropic) treatment of cell e.s.d.'s is used for estimating e.s.d.'s involving l.s. planes.
Refinement. Refinement of *F*^2^ against ALL reflections. The weighted *R*-factor *wR* and goodness of fit *S* are based on *F*^2^, conventional *R*-factors *R* are based on *F*, with *F* set to zero for negative *F*^2^. The threshold expression of *F*^2^ > σ(*F*^2^) is used only for calculating *R*-factors(gt) *etc*. and is not relevant to the choice of reflections for refinement. *R*-factors based on *F*^2^ are statistically about twice as large as those based on *F*, and *R*- factors based on ALL data will be even larger.

### Fractional atomic coordinates and isotropic or equivalent isotropic displacement parameters (Å^2^)

**Table:**

	*x*	*y*	*z*	*U*_iso_*/*U*_eq_	
O1	0.3643 (3)	0.58804 (13)	0.42377 (6)	0.0166 (3)	
O2	0.7058 (4)	0.74979 (13)	0.42394 (6)	0.0192 (3)	
O3	1.0605 (4)	0.80751 (14)	0.14662 (6)	0.0249 (4)	
O4	1.1820 (4)	1.00386 (14)	0.17463 (7)	0.0232 (4)	
N1	0.5079 (4)	0.82884 (15)	0.30422 (7)	0.0138 (3)	
N2	0.7748 (4)	1.00021 (15)	0.27284 (7)	0.0153 (4)	
N3	1.0373 (4)	0.90341 (16)	0.18170 (7)	0.0177 (4)	
C1	0.6719 (5)	0.78850 (18)	0.25230 (8)	0.0158 (4)	
H1	0.6754	0.7046	0.2332	0.019*	
C2	0.8285 (5)	0.89536 (18)	0.23422 (8)	0.0146 (4)	
C3	0.5780 (5)	0.95693 (18)	0.31538 (9)	0.0154 (4)	
C4	0.4430 (5)	1.03335 (19)	0.36860 (9)	0.0188 (4)	
H4A	0.5425	1.1183	0.3711	0.023*	
H4B	0.4712	0.9868	0.4093	0.023*	
H4C	0.2262	1.0455	0.3606	0.023*	
C5	0.3162 (5)	0.74537 (19)	0.34314 (8)	0.0163 (4)	
H5A	0.2462	0.6708	0.3170	0.020*	
H5B	0.1357	0.7945	0.3576	0.020*	
C6	0.4893 (5)	0.69604 (18)	0.40139 (8)	0.0155 (4)	
C7	0.5214 (5)	0.52893 (19)	0.47784 (9)	0.0200 (4)	
H7A	0.4783	0.5775	0.5180	0.024*	
H7B	0.7428	0.5295	0.4704	0.024*	
C8	0.4071 (6)	0.39131 (19)	0.48318 (10)	0.0237 (5)	
H8A	0.1868	0.3921	0.4889	0.028*	
H8B	0.5021	0.3489	0.5203	0.028*	
H8C	0.4582	0.3435	0.4438	0.028*	

### Atomic displacement parameters (Å^2^)

**Table:**

	*U*^11^	*U*^22^	*U*^33^	*U*^12^	*U*^13^	*U*^23^
O1	0.0165 (8)	0.0124 (6)	0.0210 (6)	−0.0005 (6)	0.0002 (6)	0.0040 (5)
O2	0.0182 (8)	0.0166 (7)	0.0228 (7)	−0.0028 (7)	−0.0031 (6)	−0.0004 (5)
O3	0.0315 (9)	0.0198 (7)	0.0233 (7)	0.0025 (8)	0.0062 (6)	−0.0028 (6)
O4	0.0218 (9)	0.0181 (7)	0.0298 (7)	−0.0029 (8)	0.0051 (6)	0.0062 (6)
N1	0.0141 (9)	0.0111 (7)	0.0161 (7)	−0.0023 (7)	−0.0004 (6)	0.0009 (6)
N2	0.0175 (9)	0.0113 (7)	0.0171 (7)	−0.0002 (7)	−0.0004 (6)	0.0006 (6)
N3	0.0188 (10)	0.0151 (8)	0.0191 (7)	0.0027 (8)	0.0007 (7)	0.0041 (6)
C1	0.0193 (11)	0.0118 (9)	0.0164 (8)	−0.0006 (9)	−0.0007 (8)	−0.0003 (7)
C2	0.0159 (10)	0.0128 (9)	0.0151 (8)	0.0010 (9)	−0.0004 (7)	0.0012 (7)
C3	0.0177 (11)	0.0093 (8)	0.0193 (8)	−0.0015 (8)	−0.0029 (8)	0.0004 (7)
C4	0.0225 (12)	0.0147 (9)	0.0193 (8)	0.0001 (9)	0.0018 (8)	−0.0004 (7)
C5	0.0144 (10)	0.0137 (9)	0.0207 (9)	−0.0033 (9)	0.0008 (7)	0.0029 (7)
C6	0.0163 (10)	0.0117 (8)	0.0184 (8)	0.0016 (9)	0.0042 (8)	−0.0009 (7)
C7	0.0219 (12)	0.0187 (10)	0.0193 (9)	0.0028 (9)	−0.0005 (8)	0.0058 (7)
C8	0.0308 (13)	0.0164 (10)	0.0239 (10)	0.0040 (10)	0.0011 (9)	0.0039 (8)

### Geometric parameters (Å, °)

**Table:**

O1—C6	1.325 (2)	C3—C4	1.482 (3)
O1—C7	1.453 (2)	C4—H4A	0.9800
O2—C6	1.199 (2)	C4—H4B	0.9800
O3—N3	1.231 (2)	C4—H4C	0.9800
O4—N3	1.224 (2)	C5—C6	1.518 (3)
N1—C1	1.364 (2)	C5—H5A	0.9900
N1—C3	1.374 (2)	C5—H5B	0.9900
N1—C5	1.452 (2)	C7—C8	1.507 (3)
N2—C3	1.317 (3)	C7—H7A	0.9900
N2—C2	1.365 (2)	C7—H7B	0.9900
N3—C2	1.431 (2)	C8—H8A	0.9800
C1—C2	1.352 (3)	C8—H8B	0.9800
C1—H1	0.9500	C8—H8C	0.9800
			
C6—O1—C7	115.04 (16)	H4B—C4—H4C	109.5
C1—N1—C3	107.79 (17)	N1—C5—C6	110.35 (17)
C1—N1—C5	124.75 (16)	N1—C5—H5A	109.6
C3—N1—C5	127.24 (17)	C6—C5—H5A	109.6
C3—N2—C2	103.97 (16)	N1—C5—H5B	109.6
O4—N3—O3	124.24 (17)	C6—C5—H5B	109.6
O4—N3—C2	118.48 (16)	H5A—C5—H5B	108.1
O3—N3—C2	117.28 (17)	O2—C6—O1	125.57 (18)
C2—C1—N1	104.11 (16)	O2—C6—C5	123.93 (18)
C2—C1—H1	127.9	O1—C6—C5	110.49 (17)
N1—C1—H1	127.9	O1—C7—C8	106.88 (17)
C1—C2—N2	112.99 (17)	O1—C7—H7A	110.3
C1—C2—N3	126.05 (17)	C8—C7—H7A	110.3
N2—C2—N3	120.94 (17)	O1—C7—H7B	110.3
N2—C3—N1	111.11 (17)	C8—C7—H7B	110.3
N2—C3—C4	125.90 (17)	H7A—C7—H7B	108.6
N1—C3—C4	122.98 (18)	C7—C8—H8A	109.5
C3—C4—H4A	109.5	C7—C8—H8B	109.5
C3—C4—H4B	109.5	H8A—C8—H8B	109.5
H4A—C4—H4B	109.5	C7—C8—H8C	109.5
C3—C4—H4C	109.5	H8A—C8—H8C	109.5
H4A—C4—H4C	109.5	H8B—C8—H8C	109.5
			
C3—N1—C1—C2	−1.0 (2)	C1—N1—C3—N2	0.8 (2)
C5—N1—C1—C2	−176.05 (18)	C5—N1—C3—N2	175.61 (18)
N1—C1—C2—N2	1.0 (2)	C1—N1—C3—C4	−179.65 (18)
N1—C1—C2—N3	179.56 (18)	C5—N1—C3—C4	−4.8 (3)
C3—N2—C2—C1	−0.6 (2)	C1—N1—C5—C6	94.6 (2)
C3—N2—C2—N3	−179.20 (17)	C3—N1—C5—C6	−79.4 (2)
O4—N3—C2—C1	−173.7 (2)	C7—O1—C6—O2	−3.9 (3)
O3—N3—C2—C1	5.9 (3)	C7—O1—C6—C5	177.17 (16)
O4—N3—C2—N2	4.7 (3)	N1—C5—C6—O2	23.4 (3)
O3—N3—C2—N2	−175.69 (18)	N1—C5—C6—O1	−157.72 (16)
C2—N2—C3—N1	−0.1 (2)	C6—O1—C7—C8	−163.26 (16)
C2—N2—C3—C4	−179.70 (19)		

### Hydrogen-bond geometry (Å, °)

**Table:**

*D*—H···*A*	*D*—H	H···*A*	*D*···*A*	*D*—H···*A*
C5—H5A···O4^i^	0.99	2.56	3.338 (3)	135
C5—H5A···N2^i^	0.99	2.56	3.509 (3)	160
C5—H5B···O2^ii^	0.99	2.39	3.175 (3)	136
C7—H7A···O2^iii^	0.99	2.46	3.362 (3)	151

Symmetry codes: (i) −*x*+1, *y*−1/2, −*z*+1/2; (ii) *x*−1, *y*, *z*; (iii) *x*−1/2, −*y*+3/2, −*z*+1.
